# A Current Sensing Biosensor for BOD Rapid Measurement

**DOI:** 10.1155/2020/8894925

**Published:** 2020-10-26

**Authors:** Yiman Liu, Jie Li, Nianxin Wan, Tianyu Fu, Lili Wang, Cong Li, Zhonghui Qie, Ao Zhu

**Affiliations:** ^1^School of Environmental and Municipal Engineering, Qingdao University of Technology, Qingdao, 266033, China; ^2^Laboratory for Marine Geology, Pilot National Laboratory for Marine Science and Technology, Qingdao, China; ^3^Jihongtan Reservoir Management Station, Shandong Water Diversion Project Operation and Maintenance Center, Qingdao 266111, China

## Abstract

In order to improve the practicality of the rapid biochemical oxygen demand (BOD) method, a highly sensitive rapid detection method for BOD that is based on establishing the correlation between current and dissolved oxygen (DO) was developed. In this experiment, *Bacillus subtilis* was used as the test microorganism, and the embedding method was used to achieve quantitative fixation of microorganisms, which could increase the content of microorganisms and prolong the service life of the biological element. The conductivity (COND) probe is used as a sensing element, so that the testing value can be read every second. In the program, the moving average method is used to process the collected data so that the value can be read every minute. National standard samples were detected to test the accuracy and stability of the method. The results showed that relative error and analytical standard deviations were less than 5%. Different polluted water was tested to evaluate its application range. The results showed that relative error was less than 5%. The results of the method are consistent with the results of the wastewater sample obtained by the BOD_5_ standard method. The proposed rapid BOD current sensing biosensor method should be promising in practical application of wastewater monitoring.

## 1. Introduction

Biochemical oxygen demand (BOD) is a widely used index for estimating the level of water pollution by organic compounds [1]. BOD can provide useful information for biological and environmental impact assessment and has been the most preferred method for environmental applications [2–5]. The standard BOD_5_ method described by APHA (1998) is the most widely used method for measuring biodegradable organic levels in waters and wastewaters [6, 7]. However, the classical BOD_5_ method is unsuitable for rapid detection and online applications [4, 8].

In order to get the BOD values within minutes, the development of a new detection method has become a very attractive option. To date, tremendous efforts have been devoted to developing new BOD detection methods to reduce assay time and simplify procedures to meet the requirements of end users [4, 7, 9–26], such as (i) biosensors based on bioluminescent bacteria, (ii) biosensors with redox mediators, (iii) microbial fuel cell (MFC) biosensor, (iv) biosensors with entrapped microorganisms, and (v) biosensors based on the bioreactor/chemist at technology [2, 5]. Among them, because of the complexity and low precision of the bioluminescence reaction, the two former technologies show the least potential in practical applications [5, 20, 22, 23, 27, 28]. Currently, the most widely adopted approach is the type of biosensor method based on the respiration rate [5, 26]. In addition, MCF has become the mostly studied method. The main issues in the development of new detection methods involve time-consuming preparation procedures [4, 12, 26], longer incubation or activation or measurement times [4, 12, 26, 29, 30], rigorous maintenance [26, 31], online applications [26, 32], and complex analysis systems [26, 33]. Although MFCs can generate stable current under constant condition, the pollution of toxic substances in MFCs usually reduces the current by affecting microorganisms [34, 35]. Membrane-type BOD sensors also have other problems, such as membrane fouling, nonportability, and insufficient online design [23, 36, 37]. Because the traditional BOD rapid monitor has a complicated pipeline system, it is difficult to clean. Residual biofilms inevitably exist in the pipeline, which will eventually affect the test results. Besides, the analytical performance of many existing rapid BOD methods is usually severely affected by the sample matrix. The measured analysis signal is highly dependent on the sample matrix, showing the inherent limitations of a narrow range of application, as well as defects in the repeatability and reliability of real sample analysis [4, 9, 12, 26, 38]. In addition, the traditional microbial immobilization technology used in rapid detection is difficult to quantify the immobilization of microbial cells. In the actual detection, the change in the number of microorganisms affects the detection results. In fact, the rapid BOD sensor achieved rapid monitoring at the expense of sacrificing unique advantages of the BOD5 method [4, 12, 26]. China advocates defining standard water quality less than 10 mg L^−1^ BOD. In order to assess such a low BOD value, building an accurate BOD monitoring system has become an important factor for monitoring the water quality [39]. The development of a new BOD method that can achieve rapidity (the inherent advantage of fast BOD sensors), solve the shortcomings of the traditional biofilm method, and maintain the widespread application of the BOD_5_ method is of an important practical issue and scientifically challenging.

In this study, we aimed to develop a simple and accurate BOD monitoring method, which can be used for real-time detection and online monitoring. According to the traditional fast monitoring method, BOD can be calculated from the reduction of DO based on the output signal of the DO sensor and the correlation between the concentration of the target substrate and BOD [40, 41]. Based on this principle, we have studied the monitoring objects and determined the method of evaluating BOD by detecting changes in electrical signals. With this detection method, the complex piping system in the instrument can be removed, the complicated cleaning work can be reduced, and the instrument volume can be greatly reduced. This method can improve the sensitivity and stability of monitoring to a certain extent and can reduce the influence caused by the unstable substrate matrix and monitoring signal. In addition, in this method, an embedding technique is used to quantify and immobilize microorganisms. The polymer matrix used to fix microbial cells makes microbial spherical particles an identification element for biodegradable organic matter, which can reduce the impact of problems such as biofilm clogging on monitoring.

## 2. Experimental

### 2.1. Method

Based on the principle of the standard detection method, the detection time is greatly shortened and the operation process is simplified. Immobilized microbial cell particles are used as reaction elements, which can realize the full mixing and reaction of microbes and water samples. In the closed reaction vessel, microorganisms decompose organic matter and consume oxygen, which results in a decrease in the concentration of DO in the water; at the same time, the value of COND will change. This study explored the reaction relationship between COND and DO, and multiple relationship curves were established. In the actual detection process, BOD can be accurately detected by detecting the change of COND in the sample and performing relevant calculations. In addition, in order to adapt to different testing requirements, this study determined a variety of testing modes.

Because the COND value can be detected every second, in principle, this method can detect the BOD value once per second. In order to ensure the accuracy and stability of the detection, the moving average calculation method is executed during the calculation process, so the BOD value can be obtained every minute.

### 2.2. Control System

First, the conductivity signal is collected by the COND probe (COND value can be detected every second). After that, the signal is collected to the transmitter through the MODBUS protocol, and the transmitter transmits the signal to the controller by the 4-20 mA signal. The controller performs moving average processing on the conductivity signal, and the data is displayed once every minute after the moving average processing. After reaching the specified running time (manual setting), the test results and reaction time will be output for the data processing and comparison.

### 2.3. Materials and Tools

PVA (polyvinyl alcohol), sodium alginate, xanthan gum, NaCl, glucose, and glutamic acid were purchased from Beijing Chemical Reagents Company. Unless otherwise indicated, all reagents used in this study were laboratory-grade materials, and all solutions were prepared with high-purity deionized (Milli-Q) water.

The BOD standard solution (136.4 mg glucose and 136.4 mg glutamic acid are dried at 103°C for 2 hours, dissolved, and diluted to 100 mL, GGA) was prepared according to the standard method. This solution in this study has a known BOD value of 2000 ± 160 mg L^−1^. And lower concentration BOD solution was prepared by appropriate dilution with deionized water.

Phosphate buffer solution (PBS, 5 mM, pH 7.0) was prepared in accordance with standard methods using diluting samples and activating and washing microbial spherical particles.

Based on theoretical principles, an experimental R&D platform and a simple prototype have been produced. The R&D platform is used for curve research and correction.

**Figure 1:**
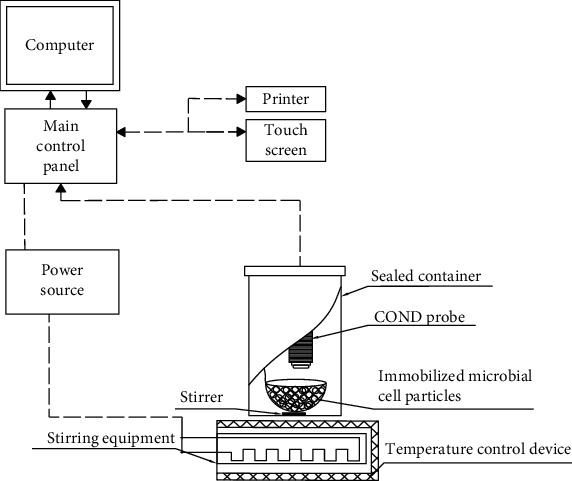
Schematic diagram of the current sensing biosensor for BOD.

**Figure 2:**
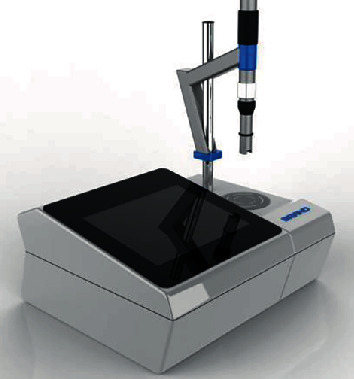
The appearance of the prototype.

The prototype is used for water sample monitoring. The structure diagram of the prototype is shown in Figure 1, and the appearance of the prototype is shown in Figure 2.

### 2.4. Immobilized Microbial Cell Particles

*Bacillus subtilis* used in the experiment was isolated from the water sample of the aeration basin of the Shandong Taian Wastewater Treatment Plant and stored at 4°C. Physiological saline solution with a mass and volume fraction of 0.8% was prepared and transferred to an Erlenmeyer flask for sterilization.

The microbial consortium was harvested and washed twice with physiological saline solution by centrifugation (6000 rpm for 10 min) at room temperature. The supernatant liquid was then discarded, and the remaining sediment was quantified by weight for construction of microbial spherical particles. Polyvinyl alcohol, sodium alginate, and xanthan gum in a mass ratio of 1 : 1 : 2 (PVA mass is 2 g) were dissolved in 30 mL of deionized water and then cooled to 40°C, then mixed thoroughly with 1 gram microbial consortium (the concentration of cell is about 3.0 × 10^9^ cells g^−1^).

The resulting mixture was placed in a sterilization mold and cooled to form spherical beads. The pellets were washed with physiological saline, then dried at 4°C for 24 hours, and stored at 4°C before use.

### 2.5. Determination of the Relationship Curve

The BOD standard solution as the experimental object was prepared according to the standard method. The BOD standard solution with a certain concentration, KCl electrolyte solution, and microorganism-embedded particles were added to the closed reaction container for full reaction. The temperature and stirring frequency remain unchanged during the reaction. During the process, the COND and DO values in the solution were detected and recorded. There are three concentration ranges of high (800+ mg L^−1^), medium (400-800 mg L^−1^), and low (400- mg L^−1^), and each concentration range is divided into different concentration gradients to conduct exploration experiments, and each group of experiments carries out multiple sets of repeated experiments. High-fitting curves were selected for the verification test.

The concentration gradient was subdivided within the detection range of each curve. The corresponding concentration standard sample is detected, and the test is repeated multiple times. Then, the deviation degree of each relationship curve is detected, and the curve coefficient is calibrated.

### 2.6. Measurement Procedures

A COND probe (DJS-1D, Shanghai, China) purchased from Shanghai Yidian Scientific Instrument Co., Ltd. was used as the transducer, which was plugged in the center of the reactor. The microbial spherical particles are placed in the reactor, and electromagnetic stirring is used to ensure that they fully react with the water sample. A temperature control system was placed at the bottom of the reactor which was used to control the reaction temperature to 25°C.

Before measurement, about 500 mL of 0.005 mol L^−1^ PBS was poured into the activation box. Then, about 2 g glucose and 0.5 g glutamic acid were added to the PBS solution which was used to provide nutrients to the microorganisms in the microbial spherical particles. The amount of microbial spherical particles used in one test is about 5 g. Then, the activation box needed to be covered, and the temperature of the activation box needed to be controlled at about 35°C (suitable for microbial growth) for 48 hours.

BOD standard solution was used to regulate the BOD sensor. The probe needs to be washed with deionized water before each monitoring. The measurement of the conventional BOD_5_ value was carried out by the standard method [6, 7]. In order to analyze the accuracy and stability of this monitoring method, the monitoring results obtained by this monitoring method need to be compared with the results obtained by the BOD_5_ method.

## 3. Results and Discussion

### 3.1. GGA Standard Solution

**Figure 3:**
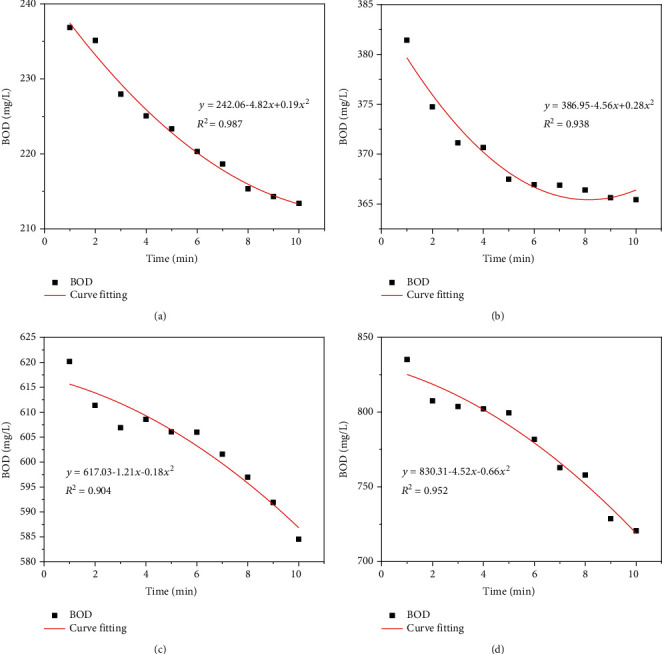
Testing results of glucose-glutonic acid solution: (a) 200 mg L^−1^; (b) 400 mg L^−1^; (c) 600 mg L^−1^; (d) 800 mg L^−1^.

In order to carry out preliminary tests on the stability and accuracy of the method, continuous tests were conducted on the different concentrations of GGA standard solution in the laboratory. This experiment was conducted on the original R&D platform. This experiment sets the concentration gradient to 200. A total of four samples with different concentrations were selected for testing. Six consecutive experiments were conducted on samples with concentrations of 200 mg L^−1^ and 400 mg L^−1^, respectively. Three consecutive experiments were carried out on samples with concentrations of 600 mg L^−1^ and 800 mg L^−1^. Due to the unstable initial reaction, the experimental data discarded the continuous data for the first 5 minutes and recorded the continuous monitoring data for 6-16 minutes. The average value of 6 sets (or 3 sets) of data corresponding to each minute is obtained. The results are shown in Figure 3. The average BOD values, deviation, and error values are shown in Table 1.

Due to some accidental errors in solution preparation and dilution, the relative deviation of different batches of experiments will be different, and the single data deviation phenomenon that occurs accidentally in multiple sets of experiments cannot be excluded. The experimental data is evenly distributed, and the data fit is high, indicating that the detection method has good stability.

The error of the test result of the sample with a concentration of 200 mg L^−1^ is relatively large. The error value of the test results of other concentration samples is within the allowable range (relative error ≤ 8%). The average deviation value also indicates that the test has better stability. In the subsequent prototype production, the curve correction work was carried out, basically solving the problem of large error in the detection results of the low concentration in this part.

### 3.2. Repeatability and Deviation

The repeatability is an important parameter that can be used to indicate the precision of the prototype. The same sample is tested under the same conditions, and the error is performed to analyze the precision of the prototype. In this experiment, five standard samples with different mass concentrations were selected for detection in each applicable range, and each concentration sample was subjected to 6 repeated tests. The average values are shown in Table 2.

The analysis of the experimental data and the significance test show that the measured value of the prototype is not significantly different from the guaranteed value of the standard sample (*p* < 0.105), and the relative error is less than 5%, indicating that the detection method has high test accuracy.

### 3.3. Stability

The stability indicates the stability of the prototype which is used to detect water samples continuously. In this test, the influent and effluent water samples of a sewage treatment plant were detected 6 times within 2 days. The prototype was set to standby except working time. The results are shown in Table 3.

The measurement results show that the average deviation of the prototype is 4.7% for the inlet water sample and 5.7% for the effluent water. The results suggest that the prototype was stable during the practical application.

### 3.4. Actual Detection

The ultimate purpose of testing various performances of the prototype is to explore the feasibility of the prototype for the detection of actual wastewater samples. In this experiment, in order to verify the feasibility of this method for testing actual water samples, *Bacillus subtilis* was used to make microbial particles to test several typical actual water samples. Each water sample was tested three times, and the average value was taken. At the same time, the BOD_5_ standard method was used to detect each water sample, and the test results were compared with the prototype test results.

The significance test indicated that the BOD mass concentration value measured by the prototype was not significantly different from the BOD_5_ standard method (*p* < 0.105). Taking the test results of the BOD_5_ standard method as the standard, the deviations of the test values of the prototype are all less than 5%. It is proved that this method and the BOD_5_ standard method have a good correlation with the test values of actual water samples. It shows that the prototype has accurate detection results for a variety of wastewater and meets the standard requirements. It can be concluded from Table 4 that the BOD values of the prototype are lower than the BOD values of the BOD_5_ standard method. Because the BOD_5_ standard method has a long reaction time, the microorganism can degrade most of the biodegradable organic compounds in the water sample, while the microorganism in the current sensing biosensor method can only quickly degrade some organics that are easily degraded in the water sample. Therefore, the test results of the same water sample by the current sensing BOD_5_ standard method are not exactly the same. Table 4 indicates that there is a large error between the test results of the BOD_5_ standard method and the current sensing biosensor method of the printing and dyeing wastewater. The reason for the result may be that the printing and dyeing wastewater contains more heavy metal ions and has a certain toxic effect on the immobilized microbial cell particles.

## 4. Conclusion

This method improves the traditional rapid monitoring instruments, reduces the complicated piping system, and optimizes the immobilization method of microorganisms. This method does not require elution. Compared with the traditional biofilm method, it can achieve quantitative immobilization of microorganisms, which can increase the number of fixed microorganisms by several orders of magnitude and control the number of the microbial cell particles used in the detection. Therefore, this method was more accurate and effective for BOD measurement which can greatly shorten the reaction time and simplify the operation steps. The test results of actual water samples show that BOD values of this method are consistent with the BOD values of the BOD_5_ standard method (error < 5%). The results of this study indicated that this method can meet the requirement of BOD rapid measurement. Toxicants such as heavy metal ions and organic toxicants have toxic effects on *Bacillus subtilis*; therefore, antitoxic strains need to be selected for detecting specific wastewater. It provides a general direction for the follow-up of this research, that is, the use of mixed strains, and precise control of the proportion of various strains, to expand the detection range and improve the stability of the detection.

## Figures and Tables

**Table 1:** The results of GGA standard solution.

Standard sample guaranteed value	Average BOD (mg L^−1^)	Relative mean deviation (%)	Maximum relative deviation (%)	Error (%)
200 ± 16	223.0	3.0	6.2	11.5
400 ± 32	369.4	1.0	3.3	-7.6
600 ± 48	603.4	1.3	2.8	0.6
800 ± 64	778.0	3.8	7.1	-2.5

**Table 2:** The results of standard sample BOD mass concentration.

Sample no.	Average BOD (mg L^−1^)		Error (%)
	Prototype measured value	Standard sample guaranteed value	
1	169.5	170.0	-0.3
2	106.8	107 ± 9	-0.8
3	10.2	10.5 ± 2.4	-2.5
4	598.3	600 ± 49.7	-0.3
5	997.8	1050 ± 87	-0.2

**Table 3:** The results of continuous monitoring.

Experiment no.	BOD (mg L^−1^)	
	Influent	Effluent
1	1140.2	130.1
2	1089.5	141.1
3	1020.4	125.8
4	1001.9	119.6
5	1067.5	120.1
6	1123.2	128.1
Average	1073.8	127.5

**Table 4:** The results of typical samples.

Sample	Average BOD (mg L^−1^)		Error (%)
	Prototype	BOD_5_	
Domestic sewage	98.3	101.3	-3.0
Printing and dyeing wastewater	99.1	109.7	9.7
Gray water	73.9	75.1	-1.5
Industrial wastewater	248.3	261.4	-5.0

## Data Availability

All data generated or analyzed during this study are included in this published article and its supplementary information files.
